# *“It always gets pushed aside:”* Qualitative perspectives on puberty and menstruation education in U.S.A. schools

**DOI:** 10.3389/frph.2022.1018217

**Published:** 2022-10-21

**Authors:** Margaret L. Schmitt, Caitlin Gruer, Christine Hagstrom, Nana Ekua Adenu-Mensah, Azure Nowara, Katie Keeley, Marni Sommer

**Affiliations:** ^1^ Mailman School of Public Health, Columbia University, New York City, NY, United States; ^2^ Office of Clinical and Community Trials, Ann & Robert H. Lurie Children's Hospital of Chicago, Chicago, IL, United States

**Keywords:** menstruation, menstrual health, puberty, adolescents, menstrual equity

## Abstract

Adolescent girls in the U.S.A. often lack sufficient education on pubertal and menstrual health topics. This educational gap may be growing given the current decline in American elementary and middle schools' delivery of sexual health education. Furthermore, little is known about the actual scope and quality of existing menstruation and puberty education in U.S.A. schools. This paper provides insights into some of the challenges with the delivery of menstruation and puberty education in schools. Qualitative and participatory research methodologies were utilized with Black and Latina girls ages 15–19 and adults working with youth in three U.S.A. cities (Chicago, Los Angeles, and New York City), exploring experiences of menstruation within school and family contexts. Findings revealed tension between school responsibility and family authority in providing menstruation and puberty education in schools, school- and teacher-related delivery challenges, and inadequate and disengaging menstruation and puberty content. Further research is needed on the effectiveness and best practices for providing this education in schools, including improved understanding on student and parent preferences, delivery mediums and the scope of content.

## Introduction

Evidence highlights that adolescent girls growing up across the United States of America (U.S.A) often lack sufficient education on puberty and menstrual health topics (1–4). This educational gap is only growing, demonstrated by recent trends indicating declines in the number of American elementary and middle schools currently delivering sexual health education (5). In the U.S.A., menstruation and puberty topics are typically embedded within the broader category of “sexual health” education programs (1). Adolescents growing up today experience puberty at younger ages than in previous decades (6), including as young as 9–11 years old (7). Despite such trends, only 21% of elementary schools, which serve students ages 8–12, currently provide puberty education (1, 8, 9). Providing menstruation and puberty education after the onset of these developmental changes can diminish the usefulness of the content for early adolescents who may socially, mentally, and physically benefit from this information (8, 10, 11). When describing the existing evidence-base, we use the term “girl” to refer to cisgender girls who experience the biological process of menstruation, while acknowledging that not all girls menstruate, and that menstruation is experienced by a broader range of gender identities.

Many girls growing up in the U.S.A. report feeling unprepared for the onset of puberty and menstruation (11). This includes a lack of knowledge on the more practical aspects of managing a period, such as how to handle menstrual cramps or how to use menstrual products (2, 4, 12). Lack of education and social support regarding puberty and menstruation have even greater implications for early maturing girls (e.g., ages 9–11 years) who report being the least prepared for pubertal changes, and are often at higher risk for poorer body image (11), depression (13–15) and engaging in risk-taking behaviors such as substance use and the early initiation of sexual activity (16, 17). A systematic review by Herbert et al. in 2016 also found that US girls who lacked information or felt unprepared for menstruation were more likely to describe having poor menarche experiences, including negative attitudes towards menstruation and menstrual distress. Improved preparation of adolescent girls on menstrual health topics was shown however to help mitigate some of the negative early pubertal timing effects aforementioned (11).

Research has shown that older adolescent girls and young women in the U.S.A. explicitly desire more information on menstruation topics, especially during early adolescence (2, 3, 12, 18). When reflecting on their early menstrual experiences in a national online survey, 165 American young women (18–37 years of age) recalled wanting more information about topics such as how to manage “menstrual accidents” (e.g., bloodstains on clothing), period cramps, and navigating menstrual product options (3). A 2021 survey conducted with 1,010 menstruating students in the U.S.A (13–19 years of age) found that 74% of students have questions about their periods, and only 43% of them report that periods were an openly discussed topic in schools (19).

Parents can sometimes act as barriers to youth receiving quality and accurate menstruation and puberty education. Evidence highlights parents' own feelings of discomfort in having conversations with their children at home on these topics (20–22). Similarly, since the 1980s, more American parents have been given the choice to opt their children out of school-led sexual health education (23–25). This practice is currently allowed in two thirds of U.S.A. states (26) and has resulted in a decline in the number of adolescents receiving sexual health education (27). Parents' personal attitudes and behaviors related to puberty and sexual health topics can be influenced by their own socio-cultural perspectives, political beliefs, and upbringing (28–30), including tendencies to regard the onset of menarche as a state of vulnerability for the initiation of sexual activity and pregnancy (31–33), rather than as a normal and healthy biological process.

A 2019 global review of puberty education programs and policies identified gaps in available evidence regarding the effectiveness of school-based puberty education in the U.S.A (1). Considering that puberty education is often packaged as part of broader sexual health education programs, it is rarely separately evaluated (1). In contrast, other elements of sexual health education programs, especially those related to reductions in risky sexual behaviors and teenage pregnancy have been more rigorously assessed (34, 35). A recent 2022 audit of every U.S.A. states' Department of Education (DOE) website found that of the 46 states with comprehensive health education standards, 85% of them included some mention of pubertal health (36). Meanwhile, the inclusion of menstruation education was more limited. Only three states (California, Michigan, and New Jersey) specifically covered menstrual product topics (36) while another three (Utah, Oregon and Michigan) included “menstruation management” in their school health standards. Such findings suggest that menstrual education, in particular, continues to be overlooked in U.S.A. schools.

This paper seeks to enhance perspectives from adolescent girls and adults in their lives on some of the challenges identified with the delivery of menstruation and puberty education in urban U.S.A. schools. This qualitative assessment highlights the tensions between school responsibility and family authority with respect to providing menstruation and puberty education. However, school- and teacher-related delivery challenges coupled with inadequate and disengaging educational content can hinder girls' ability to access appropriate and quality menstruation and puberty education in U.S.A. schools.

## Methods

### Research design

This qualitative study using participatory methods examined how school environments, families, and communities influence adolescent girls' experiences with menstruation and puberty (2). Data was collected through three methods: (1) Participatory Methodologies (PM) sessions with adolescent girls aged 15–19 (see Table 1); (2) In-depth interviews (IDI) with adolescent girls aged 15–19 and (3) Key Informant Interviews (KII) with adult actors that interact with adolescent girls, including teachers, guidance counselors, coaches, healthcare workers, and religious leaders. Older adolescent girls were purposively sampled because of their ability to reflect on their experiences of menstruation and puberty and provide recommendations for younger girls entering adolescence.

**Table 1:** Participant sample.

Type	Chicago	Los Angeles	New York City	Total
Number of PM Groups (3 sessions each)	2	2	3	7
Number of girls in PM groups	20	21	32	73
IDI	2	3	7	12
KII	8	8	7	23

### Research setting

This qualitative assessment was conducted in the three most diverse and populated American cities: Chicago, Los Angeles (LA), and New York City (NYC) (37). These cities, home to three of the largest school systems in the U.S.A. (38), experience significant funding challenges and inequalities, especially across demographic and racial lines (39, 40). Evidence suggests that schools in low-income, urban areas of the U.S.A. have difficulty affording updated textbooks, curricula, and technology and often have less experienced teachers and larger class sizes (41). The ramifications of these inequalities are vast, with evidence showing that resource limitations can negatively impact students' academic achievement and attainment (42).

### Sample and recruitment

For this study, 73 adolescent girl participants (15–19 years old) were involved in PM sessions across the three cities (see Table 1). IDIs with 12 adolescent girls were conducted using a semi-structured questionnaire. All adolescent participants (IDI and PM groups) were recruited directly through school administrators or teachers from public school institutions, or through local youth-serving non-profit staff. Schools and non-profits utilized a range of recruitment strategies to gather youth including word-of-mouth, flyers and email correspondence. The eligibility criteria provided to partnering schools and nonprofits included adolescent girls ages 15–19 who were willing to discuss issues related to menstruation and puberty. Purposive sampling of adolescent girls was used to ensure a diversity of experiences. Considering that participants came from a range of schools in each city (current and previously attended), they were asked to share generalized observations from across their educational experiences. Participant demographics from the PM sessions were 52% Black, 44% Latina, and 4% other. Participant demographics for the IDI were 58% Black and 42% Latina. Although we acknowledge the unique lived experiences of Black and Latina girls growing up in Chicago, LA and NYC, the findings from this study cannot be attributed to individuals of a specific race or ethnicity due to the participatory data collection process utilized.

The KIIs with adults (*n* = 23) were also purposively recruited from a range of organizations and professions (see Table 2) that directly interact with low-income adolescent girls in Chicago, LA, and NYC. KIIs participants were recruited from non-profits and schools involved in the PM groups or *via* colleague-based recommendations, and thus due to their professions and/or specific expertise (e.g., school nurses, sports coaches, social workers). Although participants who experience menstruation were intentionally sampled, young people were not asked to self-report their menstruating status during the research as we did not want to inadvertently create discomfort if the young person had not publicly identified their gender identity. All the adults recruited had direct experience working with adolescent girls. The research team concluded that there was a sufficient number of KII and IDI interviews when they detected a saturation of findings across interviews and participant group types.

**Table 2:** Key information interview professional roles.

Role	Quantity
School administrator	2
School teacher	5
School counselor	4
School-based social worker	3
Non-profit staff	5
Health worker	2
Religious leader	2
Total	23

The findings shared in this paper came from the following three data sources:

1. *PM sessions*: In each city, groups of 8–10 girls were gathered in a confidential space for 3, 1.5-h sessions over a 2 to 3-week period. The PM sessions included a range of activities with the results in this paper derived from 4 specific activities (see Table 3) and fieldnotes.
2. *IDIs*: We utilized semi-structured interview guides which allowed for an in-depth examination of girls' experiences, including questions which explored how girls learn about puberty and menstruation, both at school and at home, and examined the ways in which schools are often limited in their ability to provide this education. Example questions included: Where do girls typically learn about menstruation and puberty topics? Can you describe your experiences with puberty education in school? In what ways could puberty education provided in schools be improved?
3. *KIIs*: We utilized semi-structured interview guides with the adults in girls' lives to identify perspectives on the body and development issues facing adolescent girls, including how families, schools, and other societal factors impacted girls' experiences with menstruation and their changing bodies. Example questions included: What types of challenges do adolescent girls face in accessing education on menstruation and puberty? What types of challenges do schools experience when providing puberty education to youth?

**Table 3:** Examples of participatory methods used with groups of girls.

*First period stories activity*	Girls wrote anonymous personal stories about their first menstrual experiences, including their initial reactions, how they coped, what information they wished they had known prior to menarche, and advice they have for younger girls.
*Girl-friendly schools brainstorm*:	In small groups, girls brainstormed ideas for how to make their schools more girl-friendly and period supportive, with an imaginary $1 million improvement budget.
*Design a puberty curriculum for your school:*	Girls were asked to share what they think should be taught in schools about puberty and how they think it could best be provided.
*Create a puberty education campaign:*	Girls worked together in small groups to create an education campaign about puberty and menstruation, including describing key messaging and strategies for dissemination.

### Data collection

Data collection took place from October 2018 through March 2020. Data collection was implemented by a team of three cis-gender women researchers (ages 28–36) from (*blinded*) and three cis-gender women local data collection assistants (ages 22–26) that were based in each city. All PM and IDIs were carried out in private settings (e.g., school classrooms or non-profit offices). KIIs were also conducted in confidential spaces or *via* video conferencing or phone. All data collection sessions (PM, IDIs, and KIIs) were conducted in English. IDIs and KIIs were recorded and transcribed. Parental consent was acquired for all adolescent girl participants under 18 years of age, and all adolescent girls provided assent to participate. Informed consent was acquired for all participants 18 years of age or older.

All study procedures were approved by the Columbia University Medical Center and the New York City Department of Education Institutional Review Board.

### Data analysis

The research analysis team, consisting of three research team members, reviewed all transcripts, PM drawings, and fieldnotes. Analysis was completed using Malterud's “systematic text condensation,” a descriptive and explorative method for thematic analysis (43). This approach uses sequential steps, including (a) broad impression, (b) identification of the key themes, (c) condensing the text from the code and exploring meaning, and (d) synthesizing. Key themes were identified and disseminated to the broader team for further discussion, and consensus-building.

## Results

Three key themes were identified on the challenges found with the delivery of puberty and menstruation education to adolescent girls attending schools in three large cities in the United States. This includes: (1) tension between school responsibility and family authority; (2) school- and teacher-related delivery challenges; and (3) inadequate and disengaging educational content.

### Tension between school responsibility and family authority

Teachers, school counselors, administrators, and non-profit staff all described the tensions found between families and schools regarding who is deemed responsible for providing puberty and sexual health education to youth. Many school and non-profit staff indicated that parents rely too heavily on the education system to provide education on these topics. A youth non-profit staff member and former middle school teacher in LA explained her own observations of parental discomfort with discussions on puberty:

*…It seems like a lot of times the parents were hoping the schools were talking about it or even the parents just gave very minimal information to the girls. For example, it's like… ‘okay, you're going to get your period, here's a pad’…and that's it…*

Adolescent girls acknowledged this gap as well, with many describing their parents' reliance on schools for providing this type of health information. An adolescent girl in a NYC participatory session explained: *“They [parents] don't wanna do it [talk about puberty], so they want someone else to…like school.”* Several school staff indicated that in an ideal scenario, more of these conversations would occur at home. However, the diversity of home situations, including complexities with family structures and caregiver-child relationships, made this occurrence challenging for many students. A school-based social worker KII in LA pointed out the complexities of student's home circumstances at his high school, including how these challenges only further validated the need for schools to step up in providing this education:

*…I think it [puberty education] should start in the home but I do know that everyone has different living situations so that is not always going to be ideal. So, the school should definitely offer classes on it…*

School-based staff also noted that parents' own understanding, education, and views on puberty and sexual health topics sometimes impacted the scope and way these topics are introduced within the home. This includes the factual accuracy of the content shared, and if the guidance was laden with sociocultural beliefs and in some cases, misinformation. A middle school guidance counselor KII from LA explained this challenging interplay between parents and children, speaking to his own role as a parent:

*…It [school-based puberty education] saves us as parents from having that conversation. Parents grew up in a different time, and many in different countries. Students get frustrated with their parents, so I tell them to take a step back, because their parents' schooling and beliefs may be very different…*

Adolescent girls across all three cities further validated the importance of prioritizing puberty and menstruation education in schools, citing how cultural barriers and beliefs or challenging family dynamics hindered conversations from happening at home. Many girls reported feeling too embarrassed to bring up the topic, especially when their parents did not first initiate it. An adolescent girl IDI in NYC further illuminated this point, describing how own her preferences for school-based instruction were linked to the difficulties with having these conversations at home:

*… the best way [to learn about puberty] is in school. Especially when parents be too busy to even talk…but in school, that makes me feel like it should be mandatory. Definitely they should have a health class, like every school…a lot of females don't have that type of relationship with their parents…*

The reliance on schools for the provision of puberty education also generated some unique challenges for educators, especially given the sensitivity of the content. In some cases, varying sociocultural beliefs impacted views on puberty and menstruation, and educators indicated hesitancy in the possibility of contradicting information that adolescents received from home.

Another special case of this dilemma, described across all three cities, is the belief that tampon use would result in the loss of virginity. This information was often relayed to adolescent girls directly from their mothers or other family members. These widespread beliefs were frequently cited as girls' rationale for avoiding using or even trying tampons, even when they acknowledged their potential usefulness when playing sports or swimming. A female Physical Education (P.E.) and Health teacher KII in Chicago explained her dilemma in balancing respect for parental beliefs with accurate information, especially when trying to support the menstrual needs of a student:

*… One student told me her mom didn't believe in using disposable tampons or pads… you can't cross the line of their culture and what their mom believes in, but the poor girl was in a panic; she didn't want to be in that situation. I gave her options on where she could find these products and told her that she needs to talk more about this with her mom…*

Challenges like this created a complex dynamic for some educators, especially as they did not want to see a student struggle or feel unable to manage these normal and natural body changes. Similar issues were identified with menstrual pain management, with some girls describing how parental discomfort or beliefs surrounding medication made it challenging for them to manage period pain, especially in school settings where a heating pad or other holistic treatments were not viable.

### School and teacher-related delivery limitations

Both adolescent girls and school staff across all three cities described some of the current limitations around the delivery of puberty and menstruation in schools. These included teacher discomfort, their frequent inexperience with the topics, and concerns about teachers' limited bandwidth. A school nurse KII in LA expressed her concerns about teacher capacity and comfort levels, explaining:

*… teachers have so much on their plate…I can't feel confident and really support that a teacher who is teaching math and English and science is necessarily going to know all the answers or be comfortable with it…or be able to talk to a student about those topics afterwards…*

Participants described that teacher discomfort resulted from a lack of specific training about puberty and sexual health education, coupled with the pressure of their other teaching demands. Both adolescent girls and school staff indicated that P.E. teachers were often designated to provide this education. In many schools, the P.E. teachers were described as primarily cisgender men and as “not ideal” or insufficient providers of this type of education to girls. A guidance counselor KII at a Chicago middle school observed these challenges within her own institution, *“a lot of them [P.E. teachers] are men and that is a real challenge…for example, our gym teacher just doesn't feel comfortable talking about periods.”* A middle school teacher KII in NYC explained some of the challenges that arose from her school's decision to designate a male P.E. teacher to deliver this content, including his limited expertise on the subject matter:

*… there's a really large difference between having a P.E teacher who might be very good running a class like that than teaching health where they might not be comfortable teaching that class or really have the expertise. There's not a lot of priority on staffing those positions, it is rather just having that one person who teaches everything…*

The biological sex of the instructor was described as an issue for many girls across all three cities. Girl participants indicated a general discomfort with receiving education about their changing bodies from cisgender male teachers. As one adolescent girl in a participatory session in Chicago described, *“how is a man going to tell me about that if he's never had one [a period]? All my teachers who have taught me about periods have been males.”* Girls also indicated that they were less likely to ask questions about the content when it was delivered by a male teacher, and sometimes even questioned their instructor's knowledge levels on the topic. An IDI adolescent girl participant in NYC explained *“he [the teacher] wasn't really good…he was our social studies teacher, and he didn't know the health stuff. Like I want a teacher who studied this stuff, like in college.”* Adolescent girls across all three cities described their frustrations with the delivery of this content by male instructors, often noting how the use of female teachers would make this process more comfortable for them.

Rigid and sometimes mandated teaching requirements also make it difficult for some educators to prioritize puberty education. School administrators and teachers described the pressures felt by educators to prioritize content found on state-level standardized tests. School funding and rankings can be contingent on these test scores, only underscoring their importance. One middle school counselor KII from Chicago clarified her observations on this prioritization issue:

*… Typically, the gym teacher was left doing that [puberty education] by himself and it always gets pushed aside…It's not a priority and we don't know the fidelity of the implementation. It is not closely regulated because it's not part of the testing and assessment of school performance. Although its ‘mandatory’ in state schools, we don't get dinged for not doing it…*

The lack of regulation of puberty education by school districts, in addition to frequent staff and leadership turnover within schools, were all cited as reasons for the perceived inconsistent delivery of this education in many schools. Frequently this education was seen more as an ad-hoc activity rather than an integrated component of the school's curriculum. A principal of a charter middle school in Chicago validated this sentiment when describing her school's lack of uniformity each year when providing this education, explaining:

*…We don't have something consistent in place. We've had something every year and that's fine but it's like ‘ok so what are we going to do this year?’ I would like to feel like we had something we know we are going to do every single year…*

Differences were also identified across school types. A non-profit director of a girl's dance program in Chicago, who works with girls attending a range of neighborhood public schools, further elucidated on these differences:

*… Some are receiving education at school, but it depends on the school. We have girls that know everything and then some that are like ‘OMG [Oh my god], what is that? I've never heard of that before!’ I wish it was more uniform, like a standard curriculum…*

Adolescent girls were also aware of the inconsistency with puberty education delivery in their schools; an issue that was especially problematic for early developing girls. One adolescent girl in a participatory session in NYC described how her school's inconsistent approach left her in a vulnerable position as an early menstruating student:

*… I blamed my elementary school because I didn't even know what it [my period] was. My mother never told me about it. They used to teach it in elementary, but they stopped teaching it, but I don't know why they stopped teaching it…*

Some school administrators and teachers delved into some of the challenges with introducing these topics to early adolescents, particularly younger students ages 9–11. This includes how both parental and teacher discomfort likely hinders the introduction of these topics to younger students; an issue which is caused in part by societal practices linking menstruation and puberty to sex. The absence of this education before or during the onset of puberty, however, puts many younger adolescent girls at risk for negative menarche experiences. An Chicago-based adolescent girl shared in a personal written narrative her distressing experience with her first period:

*…I told my auntie that there was something wrong, I explained…my severe stomachache and the blood on the sheets and she asked me the question: ‘Didn't nobody ever talk to you about your period?’ And my response was ‘no, I didn't know what that was’… I was only in third grade at the time…*

Although such experiences of anxiety and distress were more common amongst early developing girls, they do shed light on how the needs of early developers continue to be overlooked by schools. A former middle school teacher and assistant principal in NYC further explained her concerns with delaying the provision of puberty education until middle school (ages 11–13), arguing the importance of educating youth before these changes occur:

*… I think it has to start in elementary school…I mean kids start to understand things about their bodies and have all these questions about them very early, and so sort of waiting to talk about it as puberty is coming or already started…just doesn't really make a lot of sense…*

In response to some of these challenges highlighted, some schools have begun moving towards outsourcing the instruction of puberty education to non-profit organizations with trained professionals, such as Candor Health Education, which serves schools in the Chicagoland area. Although such strategies likely improve the standardization and quality of the content provided, some teachers and students questioned if such approaches limited students' ability to ask questions and continue dialogue once the formal instruction concluded.

### Inadequate and disengaging puberty educational content

Adolescent girls across all three cities indicated that the current state of puberty education in schools was often insufficient and disengaging. Girls described that most schools prioritized the biological explanations of puberty, rather than the more practical aspects of managing periods and other associated changes, like acne and body odors. Several girls indicated that many school-based puberty education classes prioritized the association between menstruation and reproduction. A P.E. and Health Teacher KII in Chicago reinforced this notion, explaining how: *“we connect pregnancy to periods…it is kinda like when you get your period, ‘it gets real’… that is how the education is provided to them.”* Many girls expressed frustration with this approach, citing how it overemphasized sexual risks rather than the practical concerns they faced, especially in early adolescence. For example, during a group discussion in LA, one adolescent girl expressed her desire for more explicit guidance on period products, *“we would like to know how to put tampons in…no one at this table [points to four girls] has ever tried it.”* Girls also conveyed concerns and confusion about what an average period should even look like in terms of duration, quantity of blood and the coloring of both period blood and discharge. An LA-based adolescent girl during a group session supported this sentiment, explaining that girls specifically wanted to *“know what's normal…some girls get scared when their periods are irregular…and everybody has a different body type.”* The focus on better educating girls to know what is “normal” with menstrual periods, including when they may need to visit a health provider to have something checked out, was a widely shared concern by several girl participants.

Beyond the actual content provided, many girls also described disliking the types of educational mediums used to convey puberty content. Adolescent girls across all three cities described teachers' reliance on outdated or disengaging video content for providing this information. This use of videos was also described as not always being conducive for actual dialogue on the topics covered or for asking questions. As one adolescent girl in a group discussion in LA recommended:

*…It'd be good for teachers to engage with students after watching videos, and showing them websites, like ‘where to go to learn more’…and doing actual activities with us…girls and boys need to be talking about it…*

A few adolescent girls described the videos used in their classrooms as outdated and unrelatable. In a group discussion in Chicago, an adolescent girl described a puberty health education video as *“people are in bogus clothes from the 90's…and it's just hard to relate to it.”* Another girl from that same group in Chicago further explained how the video content failed to incorporate modern menstruation practices: *“they've got new products out since then, like the [menstrual] cups… they should talk about the new stuff…girls want to be more informed about this.”* Across study locations, girls expressed explicit desires to be more informed about the range of menstrual product types available on the market, noting how such discussions rarely happened within their homes or schools.

Many adolescent girls indicated resorting to the internet to fill these information gaps. One adolescent girl during an IDI in NYC explained her reliance on online sources when entering puberty, explaining, *“Google is your best friend… I always used to do that a lot, I would just google all things I didn't know.”* Girls described utilizing the internet for addressing practical information gaps, including for topics such as managing body odors, how to handle oily skin and menstrual product disposal. One adolescent girl in Chicago clarified this practice during a group session, explaining *“if you are a little girl who just started her period you would literally search ‘how to get rid of your pad’.”* There was some debate however across girls on the accuracy of information procured from the internet. Although some girls believed the information could be trusted, others were more skeptical. During a group session, an adolescent girl in LA explained: *“I usually look at like 2 or 3 websites and then if one of them contradicts itself, I won't use it…I would like just one place to go.”* The high rates of phone ownership by the girl participants in the study made this dependence on online sources a convenient option for many girls.

## Discussion

This qualitative and participatory study provides valuable learning about some of the challenges identified with the delivery of menstruation and puberty education in urban U.S.A. schools. These findings showcase the reliance by many parents and girls on the provision of puberty education in American schools. Several school and teacher-related limitations in delivering this content were identified, including instructor discomfort and limited expertise, in addition to girls' perceptions of existing puberty lessons as inadequate and disengaging. ﻿This learning supports broader evidence from the U.S.A. regarding the dearth of existing menstruation and puberty education efforts (2, 3, 11, 19), including how its absence can foster negative developmental experiences for girls, especially around menstruation (44–46).

Our study highlighted some concerns by school faculty and staff about girls' ability to access menstruation and puberty education from their families. Parental limitations related to having menstrual health and puberty conversations with their children, including discomfort or inadequate knowledge, have been well documented (2, 3, 21, 23). Despite such findings, there continues to be major political discourse on schools' role in providing developmental health education in the U.S.A. In part, the debate seems to stem from the fact that sexual health education includes a broad range of topics from puberty to sexual intercourse and sexually transmitted diseases. As a result, menstrual health and puberty education often gets tied up in the debate about who should teach adolescents about sex and sexual health. For example, the abstinence-only education movement continues to champion parental rights and the notions that such education should be the responsibility of parents as opposed to schools. In contrast, comprehensive sex education proponents call for schools to play a larger role in providing this education (47, 48), citing the broad evidence base describing parental discomfort, tendencies for misinformation, and reluctance to initiate conversations (11, 23, 29, 49).

While this debate continues, this study found that many school staff and girls felt that parents frequently depended on schools for providing puberty education. This aligns with other sexual health education research conducted in the U.S.A., including a 2020 qualitative survey conducted with 484 parents living in South Carolina (50). This study found that many parent respondents viewed the provision of sexual health education as an “obligation” of public schools to their students (50). Notably, evidence also suggests that support for school-led sexual health education receives strong bipartisan support from parents across the political spectrum in the U.S.A. (30), suggesting that such policy debates may be out of sync with public sentiment.

Our findings showed that many adolescent girls had not received puberty education by the time they reached menarche, a situation that may be becoming more common as the age of menarche decreases in the U.S.A. Recent data indicates that 10% of American girls reach menarche by age 10 (51); however, sexual health information is often not provided in elementary schools, especially prior to 5th grade (ages 10–11) (1). Because of varied political and religious beliefs in the U.S.A., the laws regarding the provision of sex education vary widely state to state. Puberty education is often included in comprehensive sexual health education guidelines (52), but when some parents hear the term “sex education”, they assume the conversations will be about sex. This appears to be less of an issue with the content of puberty education itself, and more of an issue of framing. Separating puberty education from sex education may help assuage parental discomfort, reduce the number of parents who opt their children out of these lessons, and allow schools to provide this information to younger adolescents before the onset of puberty without facing parental backlash. Additionally, adolescents in our study described discomfort about the association between puberty, womanhood, and sex. The separation of puberty and menstruation education from sex education could provide adolescents with the information they want about their developing bodies, while breaking down the stereotypes linking the onset of puberty to womanhood, pregnancy, and sexual intercourse.

Overall, this study found that many girls desired more education on menstruation and puberty topics in schools, including more emphasis on practical guidance. Broader evidence supports this sentiment, including strong preferences that schools not only cover these topics but formalize it within the curriculum. A 2021 survey of 1,010 menstruating students (13–19 years of age) across the U.S.A found that 73% of respondents believed the menstrual health education should be part of the core curriculum, much like math or other key subjects. Only 5 states currently specify that menstrual products or menstruation management should be included in their DOE Health Education Standards (36). Although the inclusion of menstruation education within state-level policies is important, research suggests a frequent disconnect between state and local school district requirements, including a significant lag time regarding the uptake of new measures (53).

School staff and adolescent girls identified a series of institutional limitations to the provision of menstruation and puberty education. This includes issues with the lack of training of teachers, instructor discomfort with the content, and concerns about the biological sex of the instructors. Teacher discomfort or low-confidence with instructing on these topics has been well-documented in the literature (54, 55). Studies have described that teachers are often reluctant to even discuss menstrual topics (55, 56) and may present period topics in a stigmatizing manner (57); which can perpetuate negative perceptions of menstruation as shameful and requiring discretion (57, 58). These findings highlight the need for instructors to be reflective about their own experiences with menstruation, especially when considering teacher education models (58). A 2022 survey of 789 teachers in the United Kingdom found that 80 percent of teachers felt that training would benefit their menstrual education instruction practices (59). Some research has documented the actual value of in-service training, including improved confidence while navigating these topics in the classroom (58, 60).

Our findings indicated that the prioritization of puberty and menstrual health topics was sometimes challenging for schools and individual teachers. Such findings are not surprising given that broad instructional competencies required by many U.S.A. teachers. For example, many P.E. and health teachers are often responsible for covering a range of topics beyond puberty including mental health, nutrition and drugs and alcohol. In response, some schools have begun to depend on external organizations or educators to support the provision of this education (61). Benefits of relying on outside experts include that they may have access to a higher quality curriculum, and if they are working with an entire school district or county, they can ensure greater consistency in messaging across schools.

However, there are some challenges with outsourcing the delivery of this education, including cost considerations for already resource-stretched schools and districts. Another potential issue is that external educators often have very limited time in the classroom, so students may be left with questions after the session and no one to turn to for answers. Some students, however, may feel more comfortable asking questions about sensitive topics to an outside educator rather than their teacher who they have to interact with every day (62). Notably, parental concerns with some external organizations have also been documented. Planned Parenthood, for example, is currently one of the most used external organizations to provide puberty and sexual health education in schools. In 2019, parents from a LA school pushed back on the use of Planned Parenthood for providing this health education due to their discomfort with the organizations broader role in providing abortion care (63). Further research should be done to examine the benefits and drawbacks of using external actors for providing this education, including student and parental preferences and the cost-effectiveness of such approaches.

This study found that many adolescent girls considered the menstruation and puberty education provided in schools to be of poor quality, often limited in scope and disengaging. This led many girls to depend on online sources to address these information gaps. A 2016 digital survey with U.S.A. teens ages 13–18 years support these findings, where 84% of respondents reported receiving health information online, including a quarter of them receiving “a lot” of information from these sources (64). Recognizing these trends, many youth health organizations have developed tailored and engaging puberty content designed to be accessed on online platforms like YouTube and TikTok (65–67). The provision of online content offers an avenue to easily reach a wide range of adolescents with convenient and free resources. While this content can help address the gaps in knowledge, research does suggest that they may not necessarily change adolescent attitudes towards puberty (68). This limitation further highlights the importance of parents and schools having dialogue with their children about both the physical and emotional sides of puberty and instilling in them that puberty and menstruation are healthy, normal aspects of growing up.

## Limitations

There are a few limitations to note for this study. First, these findings focused on low-income adolescent girls living in three select cities in the U.S.A. Our findings are therefore unable to capture the breadth of experiences of girls growing up in rural and suburban communities and across different socioeconomic situations. Second, given that this study targeted adolescent girls, it cannot provide insights on the experiences of trans and gender non-conforming students and the unique puberty and menstruation educational needs that this population may have. Third, given that older adolescents (15–19 years of age) were sampled for this study, there is potential that some of the participants might have experienced recall bias when asked to reflect on their early puberty and menarche experiences.

## Conclusion

As a result of ongoing challenges in accessing quality menstruation and pubertal health education in schools and at home, many adolescent girls in the U.S.A. are under-prepared to navigate puberty and menstrual health comfortably and confidently. Four key recommendations for future research, practice and policy include: One, research is needed to identify the most effective methods for providing menstruation and puberty education in schools, including developing a better understanding of student preferences for the medium of communication and the topics that should be included; Two, school systems should provide increased attention, training, and resources related specifically to menstruation and puberty education to ensure that instructors are confident and comfortable in delivering this information; Three, improved school engagement and support targeting parents, as to ensure for more collaborative and encouraging learning experiences for students; Four, puberty and menstrual health education should be more effectively standardized and monitored at the state and district-levels to ensure improved accountability and implementation fidelity. Ultimately, both improved evidence and prioritization of menstruation and puberty health education by public school systems will ensure more equitable access to this critical information by all adolescents in need growing up across the U.S.A.

## Data Availability

The raw data supporting the conclusions of this article will be made available by the authors, without undue reservation.
